# Feasibility of Ski Mountaineering for Patients Following a Total Knee Arthroplasty: A Descriptive Field Study

**DOI:** 10.3390/ijerph16091582

**Published:** 2019-05-06

**Authors:** Simon Haslinger, Daniela Huber, David Morawetz, Cornelia Blank, Johanna Prossegger, Tobias Dünnwald, Arnold Koller, Christian Fink, Arnulf Hartl, Wolfgang Schobersberger

**Affiliations:** 1 Institute for Sports Medicine, Alpine Medicine and Health Tourism, University for Health Sciences, Medical Informatics and Technology, 6060 Hall/Tirol & Tirol-Kliniken GmbH, 6020 Innsbruck, Austria; simon.haslinger@tirol-kliniken.at (S.H.); david.morawetz@umit.at (D.M.); cornelia.blank@umit.at (C.B.); tobias.duennwald@umit.at (T.D.); arnold.koller@tirol-kliniken.at (A.K.); 2 Institute of Ecomedicine, Paracelsus Medical University Salzburg 5020 Salzburg, Austria; daniela.huber@stud.pmu.ac.at (D.H.); johanna.prossegger@pmu.ac.at (J.P.); arnulf.hartl@pmu.ac.at (A.H.); 3 Department of Physiotherapy, Salzburg University of Applied Science, 5412 Puch/Urstein, Austria; 4 Gelenkpunkt—Sports and Joint Surgery, 6020 Innsbruck, Austria; c.fink@gelenkpunkt.at; 5 Research Unit for Orthopaedic Sports Medicine and Injury prevention, Institute for Sports Medicine, Alpine Medicine and Health Tourism, University for Health Sciences, Medical Informatics and Technology, 6060 Hall, Austria

**Keywords:** total knee arthroplasty, total knee replacement, return to sport, ski mountaineering, muscle fatigue

## Abstract

*Background:* Total knee arthroplasty (TKA) is socially relevant due to its high prevalence, high incidence and the affected population. A subpopulation of TKA patients exists that strives to be active and also return to sports after total joint replacement. In this context, a further group of TKA patients is interested in high-impact physical activities and want to proceed with such activities even after surgery. Focusing on winter sports, there is still a lack of evidence on whether ski mountaineering is feasible for this subgroup of patients. Therefore, this feasibility study examines the effects of moderate ski mountaineering on strength, balance, functional abilities and mental health in persons following a TKA. *Methods:* Eight patients (six males, two females; median age, 63 ± Interquartile range 9 years) with TKA were included in this study. The volunteers, who were pre-selected for a 7-day holiday in Sankt Johann (Tyrol, Austria), participated in five guided ski mountaineering tours. Statistical analyses of non-parametric longitudinal data were performed using analysis of variance. For gait parameters and the Feeling Scale, one-factor longitudinal models were used. Statistical significance was set at the level of *p* < 0.05. *Results:* A significant decrease in the S3-Check MFT stability index (*p* = 0.04), a significant increase in general well-being (*p* = 0.05), and a trend towards a decrease in general stress (*p* = 0.1) were detected, while all other parameters were unaffected. *Conclusion:* A 7-day recreational ski mountaineering holiday had no negative effects on ski-experienced patients with TKA and seemed to increase well-being. Further studies should focus on larger groups and use controlled designs. Additionally, long-term effects should be evaluated.

## 1. Introduction

End-stage joint deterioration due to osteoarthritis (OA) is the most common reason for patients to seek a total joint replacement, to relieve pain and increase quality of life [1,2]. The Age, Gene\Environment Susceptibility-Reykjavik Study (AGES Reykjavik Study) showed a prevalence for at least one joint replacement operation due to osteoarthritis of 13.6% and an annual incidence of 1.4% over a five-year follow-up period. The prevalence of total knee arthroplasty (TKA) was 5.2% for males and 6.7% for females, whereas the incidence of TKA was 2.9% for males and 3.8% for females [3]. In Austria, the number of knee replacements per 100,000 population increased by about 70% between 2002 and 2014 [4]. Physical activity is not only recommended for improving bone quality and prothesis fixation after total joint replacement, but also due to its positive effect on general health, disease prevention and physical and mental well-being [5,6]. Furthermore, outdoor sports are mentioned to have benefits concerning not only physical and mental outcomes, but are also assumed to improve social development for individuals and groups [7]. However, in a total joint replacement, there is a mismatch between surgeons’ and patients’ expectations in regard to physical activity, and some physicians tend to restrict patients’ ambitions to return to risky sports [8]. In fact, recommendations exist regarding low-impact sports but are rare concerning high-impact sports, such as alpine skiing or ski mountaineering. However, a previous study on sports activities after TKA stated that 70% of the participants performed alpine skiing 24 months after surgery [9].

It is also well-documented that physical activity can lead to muscle fatigue and a decrease in body stability and balance, and hence potentially can increase injury risk [10]. A decrease in quadriceps and hamstring strength, as an indicator of muscle fatigue, is obvious even after 1 day of alpine skiing [11]. Furthermore, as there are distinctive underlying factors involved in muscle fatigue (i.e., changes in different systems, e.g. nervous, vascular, and energetic), the overall consequence is a decrease in muscle strength; therefore, muscle fatigue can be defined as an exercise-induced decline in the ability of muscles to produce force and power [12].

Concerning skiing and TKA, recent studies evaluated the effects of alpine skiing on strength and gait symmetry. In this context, alpine skiing was recommended, despite being categorised as a high-impact sports activity, in order to reduce strength deficits, improve gait performance and increase balance ability [13,14].

However, there is currently no research in the field of ski mountaineering. Ski mountaineering as a leisure winter sports activity is enjoying increasing popularity, especially in the Austrian Alps [15]. Though, in contrast to alpine skiing, which is largely characterised by multiple passive ascents (via cable cars) and active descents on pre-prepared slopes, ski mountaineering consists of a single active ascent and one descent in open terrain under varying environmental conditions, and therefore might be more demanding. Therefore, similar to alpine skiing, ski mountaineering has a certain impact on muscle fatigue and has to be considered as high-impact sport for patients with TKA as well. In a recent study on ski mountaineering with healthy subjects, participating in a week of consecutive ski tours, we found that muscle strength was decreased already after the first ski tour and remained at a lower level without any cumulative effect during the time course of the week [16]. Similar to alpine skiing, ski mountaineering can be considered as a high-impact sports activity; however, evidence-based recommendations for patients with a TKA are still lacking.

Thus, the aim of this feasibility study was to evaluate whether TKA patients, experienced in ski mountaineering prior to and after surgery (engaging in recreational ski mountaineering once and repeatedly over a time period of a 1-week holiday), show muscle fatigue (expressed as a decrease in muscle strength), increased gait asymmetry and a decline in balance performance. In addition, the impact of this sport on mental health was also examined. As a pilot study, this investigation should also provide a basis for further studies with larger numbers of cases, provided that there is an absence of adverse effects.

## 2. Materials and Methods

### 2.1. Subjects

Patients with TKA were recruited directly via trauma centres, as well as via digital print media adverts in local newspapers in Tyrol and Salzburg (Austria). The inclusion criteria were: unilateral TKA (operated on between 2008 and 2016), age between 40 and 70 years, above average skiing proficiency based on a score between 6 and 9 on a visual analogue scale (VAS) for skiing (0: “no skiing skills” to 10 “excellent skiing under all terrain and snow conditions”), experience in ski mountaineering in open terrain before and after TKA, availability for a preliminary and a 14-day follow-up examination, a 1-week recreational ski mountaineering holiday, an exercise capacity of more than 110% (age-predicted) based on a cycle ergometry (Lode B.V., Groningen, Netherlands; for details, refer to Koller et al. [11]) and medical approval based on a preliminary medical and orthopedic examination. These medical examinations included medical history, a 12-lead electrocardiogram at rest, spirometry, blood laboratory parameters, including red and white blood cell counts, thyroid hormones, blood lipids, inflammation markers (e.g., C-reactive protein) and liver and kidney parameters, and stepwise cycle ergometry until exhaustion. Furthermore, a clinical examination of the TKA leg as well as of the contralateral leg has been performed by an orthopedic surgeon. Stability, range of motion (ROM) and symptoms have been assessed according to the Knee Society Score prior and post intervention [17]. Exclusion criteria were acute illnesses and injuries shortly before and during the investigation, chronic diseases, and pain therapy with non-steroidal anti-inflammatory drugs (NSAIDs), corticosteroids and other inflammation inhibitors. Before participants were finally enrolled in the study, they had to sign an informed consent form.

The study was conducted in accordance with the declaration of Helsinki and was approved by the Ethics Committee of the Medical University Innsbruck (EC No: 1125/2017).

### 2.2. Study Design

The trial took place during 1 week in January 2018 at the ski area of the village of St. Johann, Tyrol, Austria. Participants performed five ski mountaineering ascents and descents within 6 days, including a rest day (day 4). Additionally, participants were grouped into two performance categories (low, high) based on their exercise capacity (maximum Watts per kilogram body weight) to obtain two homogenous groups with a similar exercise load for all subjects during the intervention. Both groups were instructed about the routes and safety aspects of ski mountaineering and were accompanied by mountain guides, certified by the International Federation of Mountain Guides Associations, as well as persons providing medical support and members of the study team. Participants were also instructed to keep up with the pace corresponding to their baseline endurance capacity (below 75% of the individual heart rate (HR) reserve, calculated according to the formula of Karvonen [18]). All subjects completed the same course, including an average ascent of 2–3 hours, depending on the vertical height of the summit, with an overall duration of 4–5 hours including breaks. During the vacation, alcohol consumption was limited to an amount equal to 500 ml beer per day. The intervention process filled most of the day, and participants used the few leisure time periods for short city trips to St. Johann and were allowed to moderately use the spa area of the hotel for regeneration and relaxation.

### 2.3. Environmental Parameters of St. Johann/Tyrol

The study was performed in St. Johann in Tyrol (GPS 47°31’18.0”N 12°25’38.9”E), which is located 659 m above sea level. The ski tours were performed in the morning, to ensure similar conditions during the 1-week investigation. Whereas weather conditions were stable (sunny weather, with temperatures ranging from −8° to +5° at the starting point), snow conditions varied from deep snow to powder, corn and crusted snow, depending on the elevation and slope orientation.

### 2.4. Measurements

In order to determine the feasibility of ski mountaineering for patients with TKA, strength, balance, functional abilities and mental health were assessed.

**Figure 1:**
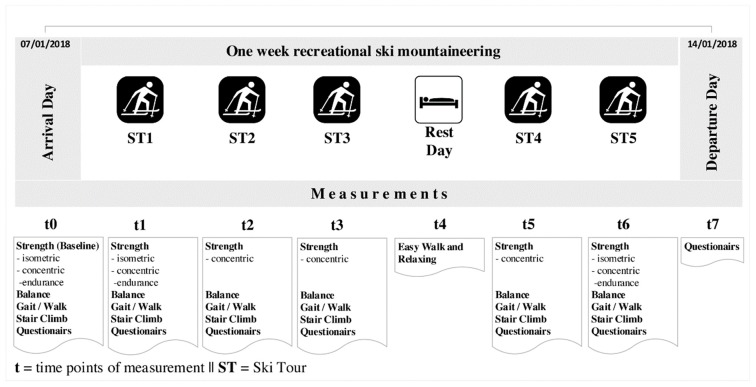
A schematic of the chronological process of the trial.

An overview of the measurement times and coding is given in Figure 1. Concentric maximum strength of the muscles of interest, static balance, and functional abilities were assessed at t0, t1, t2, t3, t5, t6 (t0…t6 = timepoints of measurement). In addition, isometric strength and concentric strength-endurance of the extensor and flexor muscles of the thigh muscles were measured at t0, t1 and t6. With regard to mental health, the Feeling Scale and the Felt Arousal Scale were completed before and after each ski tour (t1, t2, t3, t5, t6). The Mood-Scale, Recovery-Stress Questionnaire (REST-Q) Sport and Short-form (SF)-12 questionnaires were completed at t0 and t7. No assessments were performed on the rest day. For each ski tour, the duration, distance and altitude were recorded. Before each ski tour, a visual analogue scale was used to determine the current state of health (−5 = very bad, 0 = neutral, +5 = very good) in order to avoid participation of those with acute health issues (t1, t2, t3, t5, t6). Post-exercise, participants filled in the Borg Scale. (t1, t2, t3, t5, t6). There were two subjects at the same time for testing, and the time difference between pairs was 15 minutes. The sequence of examinations was as follows: questionnaires–balance–gait–stair climb test–strength. Strength and functional abilities were tested between a minimum of 1 and a maximum of 3 hours post exercise, including a 20-minute warm up on the cycle ergometer.

### 2.5. Heart Rate Monitoring and Global Positioning System (GPS)-Tracking

A sports watch, in addition to a chest strap (Suunto Ambit2 R, Finland), was used to monitor heart rate (HR), ascent time, ascent altitude difference, ascent distance and GPS tracking data. These watches enabled a complete raw data export. In addition, all hiking tours were traced via GPS handhelds (Garmin GPSmap 62s, Garmin Ltd, Neuhausen am Rheinfall, Swiss) to verify the accuracy of the Suunto Ambit R watches.

### 2.6. Muscle Strength Measurements (Isokinetic Dynamometry)

To determine the maximal voluntary concentric and isometric muscle strength of the quadriceps and hamstrings, isokinetic dynamometry was performed using a HUMAC NORM Testing and Rehabilitation System (Proxomed Ltd., Alzenau, Germany). After warming up for 20 minutes on a bicycle ergometer (60 Watts females, 80 Watts males), participants were seated on the dynamometer with their hip flexed to approximately 90° and their trunk secured with dual cross-over straps and a waist strap. The range of motion at the knee was set between 0° (fully extended) and 90° (flexed). A thigh strap on the leg to be tested was used to fix the thigh, allowing only flexion and extension. The testing protocol consisted of concentric and isometric quadriceps and hamstring contractions. For familiarisation with the testing procedure, participants performed four submaximal concentric repetitions at an angular velocity of 60°/s, followed by a 5-second break prior to five maximum concentric peak torque repetitions. For maximal isometric testing, participants were familiarised with two submaximal isometric repetitions followed by three maximal isometric repetitions. Therefore, participants were instructed to exert maximum strength within 5 seconds against the testing apparatus, to identify the best repetition as the maximum peak torque. The strength endurance test (SE) consisted of 12 repetitions at an angular velocity of 180°/s, following four familiarization repetitions and a 5-second break. The testing procedure included a 2-min rest period between each testing procedure. Only the TKA-leg was tested in each participant, which is reasonable because OA is known to be a bilateral symptomatic in many cases. Furthermore, the untreated side is considered to be subjected to a higher functional overload [19]. The test protocols were similar to those used in previous studies on alpine skiing [11,13].

### 2.7. Gait Analysis

To survey lower limb movements during gait, an analysis was performed using the Mobility Lab (APDM Inc. Portland, OR, USA), which is a validated and reliable gait and balance measurement tool [20]. The task was to travel a distance of 7 meters at a self-selected pace, turn around and go back to the starting point for another trial. This process continued for 2 minutes. The integrated OpalTM-sensors of the three cuffs gave information on cadence, double limb support, single limb support, stance and swing phases, stride length, elevation at mid swing, lateral step variability, circumduction and gait speed.

### 2.8. Balance in One- and Two-Legged Stand

To assess balance on the TKA leg, a one-legged measurement was performed using Mobility Lab (APDM Inc., Portland, OR, USA), and a reliable protocol [21]. Subjects stood on the affected leg; the other leg was lifted off the ground. The participants were instructed to hold this position for 30 seconds. As a surrogate parameter for static balance, the Sway Area (area of an ellipse covering 95% of the sway angle in the coronal and sagittal planes) was determined.

Static balance was assessed using the S3-Check MFT (Bodywork, Trend Sport Trading GmbH, Großhöflein, Austria). The S3-Check is a standard measure to identify deficits in the functional locomotor system. The participants are instructed to keep the labile disc centred for 30 seconds. This process was performed twice with a 15-second gap in-between. So, within 60 seconds, a person’s stability (stability in relation to standard data (percent)); Stability index: score 1 (very good) to 9 (very weak)), symmetry (%-deviation from horizontal plate position) and sensorimotor (sensorimotor function in relation to standard data (percent)); Sensorimotor index: score 1 (very good) to 9 (very weak)) functions are recorded. The stability index, which is the total score across all measured parameters, allows for a reliable and objective assessment of a person’s balance ability based on normative data [22].

### 2.9. Stair Climb Test

To further determine the functional abilities of the lower limbs, the Stair climb test was used. In this test, participants had to complete 10 steps of a staircase (step height 17 cm, step depth 28 cm, step width 136 cm and a pedestal considered as step 10 with depth × width of 45 × 136 cm) as fast as possible in ascending and descending directions. The handrail was fixed at the left side of the staircase (height 105 cm). The time (Brower Timing System, Draper, UT, USA) taken was measured upwards and downwards, as well for turning around at the end of the stairs and the total time. Participants were instructed to use the hand rail to increase their sense of security. This assessment shows high test–retest reliability and good inter-rater reliability [23,24].

### 2.10. Borg Scale

The 15-point Borg scale assesses perceived exhaustion (range: 6 to 20; 6 = none, 9 = very easy, 20 = maximum) [25]. This assessment was only used to determine whether participants exercised at moderate intensity.

### 2.11. Feeling Scale (FS) and Felt Arousal Scale (FAS)

The Feeling scale (FS) is a bipolar single item scale, which was used in previous studies. Eleven answer options, ranging from −5 to +5 (−5 = very bad, 0 = neutral, +5 = very good), are used to rate pleasure [26]. The Felt Arousal Scale (FAS) is also a single item scale, and has six answer options ranging from 1 to 6, with keyword entries only at 1 (low arousal) and 6 (high arousal); it is used to rate the participant’s level of activation [27].

### 2.12. Recovery-Stress Questionnaire for Athletes: Shortened German Version (EBF 24)

To obtain stress reactivity potential as a state of current strain and recovery, we used the shortened German version (EBF 24 B/3) of the Recovery-Stress Questionnaire for athletes (RESTQ-Sport) [28]. This questionnaire covers seven different dimensions of strain (general, emotional and social stress, conflicts/pressure, fatigue, lack of energy and physical complaints), as well as five subscales of recovery (success, sleep quality, general well-being and social and physical recovery) [29].

### 2.13. Short Form 12 Health Survey (SF-12)

The German version of the SF-12 was used to assess the physical and mental health state. In contrast to the SF-36, it takes less than 2 minutes to fill in and consists of 12 items (two each for physical functioning, limitations associated with health problems, limitations caused by emotional problems, and general mental health, and one each for bodily pain, general health perception, vitality, and social functioning) [30,31].

### 2.14. Mood Scale (Bf-SR)

This assessment is a multidimensional method indexing the current mood state. It is based on the basic bipolar dimension of “tension” and “assessment” [32]. The shortened German Version of the Bf-SR, used herein, consists of 24 items assessing the current psycho-physical state through contrary adjectives (i.e., undecided, decisive, neither-nor) [33].

### 2.15. Statistical Analysis

Statistical analyses were performed using R-GNU software (version 3.4.4; General Public License, R Foundation for Statistical Computing, Vienna, Austria) and SPSS software (version 25.0; SPSS Inc., Chicago, IL, USA). Statistical significance was set at the level of α ≤ 0.05 for all tests. Due to the small sample size and the data distribution, all variables are expressed as median and interquartile range (IQR) unless otherwise indicated. Longitudinal data analysis was performed using the nparLD-package [34], which allows for a fully non-parametric analysis of variance-type testing. Post hoc tests were applied in cases of a significant main effect of time. For the post hoc test, we also used nparLD and corrected the *p*-values according to Bonferroni.

For analysis of gait parameters and the Feeling Scale, we used one-factor longitudinal models (F1-LD-F1) and included group as the between-factor (unaffected versus affected leg, before and after a ski tour) and time as the within-factor (t1–t5). From this, the F-distribution, the significant main effects of time and treatment, and the time–treatment interaction could be derived.

As a measure of effect, we used the relative treatment effect (RTE). Relative treatment effects can account for outliers [35], which is of relevance given our small sample size.

In addition, correlations (Spearman’s rho) were calculated to detect possible redundancies or relationships between mental and physiological parameters in the test battery, as well as connections between tour data and the results of the other assessments. Since this was a feasibility study, in which different influencing factors of ski mountaineering performance in patients with TKA were examined for the first time, a power analysis was not undertaken.

## 3. Results

### 3.1. Demographics and Patient Characteristics

A total of 39 persons were contacted; of these, 22 fulfilled eligibility criteria and were invited to participate in this study (17 persons out of 39 were too old or too young or had a prosthesis on both sides). As a result of agreement with the initial inclusion criteria, 16 subjects have signed the consent form and were then invited for a medical examination. The most frequent causes for exclusion were pre-existing cardiovascular diseases or a lack of fitness. Finally, in this patient cohort, none of the non-operated legs were symptomatic. At the clinical examination prior to and post intervention, none of the participants complained about symptoms. In total, 11 patients were included.

The following reasons for dropout were recorded: two participants were excluded immediately before the study investigation due to an acute respiratory disease. Another person injured his ankle during a descent after a ski tour on the third day of the investigation, on ski tour 2. Since we performed a per-protocol analysis, this subject’s data were not included in the final statistics.

In summary, this feasibility study finally included eight persons following a TKA (six men and two women) ranging in age from 49 to 69 years (median age, 63 years, IQR 9), with a body mass index (BMI) of 24.67 (IQR 3.34). Four persons had their TKA on the right side, and four on the left side. The median prothesis-age was 5.0 (IQR 2.75) years. The median Knee Society Score was 95 (IQR 10) before the intervention, with no changes even at 14 days after the study investigation. The median tour weight of the subjects was 93.9 (IQR 17.33) kilograms, including the equipment (backpack, skis, ski boots) weight of 17.45 (IQR 2.23) kilograms. Regarding performance during the preliminary cycling ergometry, the following data were obtained: the median Wmax/kg was 2.85 (IQR 0.5), and VO2max (calculated by means of body mass in kg and maximum Watts [36]) was 37.55 (IQR 6.73) ml O2/min/kg. Also, there was a 75% heart rate reserve of 143 (IQR 5) beats per minute with an age-predicted exercise capacity of 144% (IQR 19.5).

### 3.2. Route Information and Exercise Load

On average, 2–3-hour ascents were performed during the study week. All participants maintained their predefined pace with respect to the average heart rate during the ascent, and remained below their individual 75% heart rate reserve. Descriptive exercise data are outlined in Table 1. The rate of subjectively perceived exhaustion on the Borg Scale showed a subjective exercise load ranging from “relatively light” (10.5, IQR 2) to “mildly difficult” (12.5, IQR 3). Values remained constant throughout the 5 days of ski mountaineering. Hence, a similar and moderate exercise load can be assumed for all participants. Correlations between tour data and other relevant results were not found; therefore, these were not included in the evaluation.

### 3.3. Strength

Analysis of variance of the concentric and isometric maximum muscle strength of the extensors and flexors revealed no significant changes over time in the extensor muscles (concentric: F(2.52,∞) = 0.87, *p* = 0.44, relative treatment effect (RTE) 0.52; isometric: F(1.54,∞) = 0.19, *p* = 0.77, RTE 0.51) or flexor muscles (concentric: F(2.64,∞) = 2.07, *p* = 0.11, RTE 0.48; isometric: F(1.53,∞) = 0.37, *p* = 0.64, RTE 0.51). Also, the maximum strength-endurance and endurance of these muscle groups showed no significant changes over time, in either the extensor muscles (maximum: F(1.93,∞) = 0.10, *p* = 0.90, RTE 0.50; work: F(1.62,∞) = 0.89, *p* = 0.39, RTE 0.51) or the flexor muscles (maximum: F(1.93,∞) = 0.59, *p* = 0.55, RTE 0.52; work: F(1.43,∞) = 0.71, *p* = 0.44, RTE 0.53). The data are shown in Table A1.

### 3.4. Functional Abilities

One-factor longitudinal analysis of Mobility Labgait parameters (cadence, double support, single limb support, stance and swing phases, stride length, elevation at mid swing, lateral step variability, circumduction and gait speed) showed no significant effects regarding time, treatment or the time–treatment interaction (results are shown in Table 2). Analysis of variance of the Stair climb test data showed no significant changes over time (F(2.03,∞) = 1.01, *p* = 0.37, RTE 0.54). All descriptive data are shown in Table A2.

### 3.5. Balance

The analysis of the S3-Check MFTstability index shows a significant time effect (F(3.22,∞) = 2.76, *p* = 0.04; RTEs 0.57, 0.60, 0.44, 0.53, 0.47 and 0.39 for t0, t1, t2, t3, t5 and t6, respectively), indicating a decrease in stability. According to the post hoc test, a significant reduction in the stability index was also present at t6 (F(1,∞) = 6.67, adjusted *p* = 0.05). The S3-Check MFTsymmetry index (F(2.71,∞) = 0.55, *p* = 0.05, RTE 0.63), the symmetry index, presented as the % deviation from the middle (F(4.05,∞) = 0.84, *p* = 0.5, RTE 0.37) and sensorimotor function (F(2.88,∞) = 0.36, *p* = 0.77, RTE 0.53) showed no significant changes over time. Analysis of variance of the Mobility Lab Sway area revealed no significant changes over time (F(2.95,∞) = 0.13, *p* = 0.94, RTE 0.48). The data are shown in Table A1.

### 3.6. Mental Health

A correlation analysis of the Recovery-Stress Questionnaire subscales was performed and indicated very high correlations between certain subscales. Thus, it appears that these subscales measure similar characteristics, which is why only three subscales were included in the final analysis to avoid multicollinearity. The analyses revealed a significant increase over time in general well-being (F(1,∞) = 3.9, p = 0.05, RTE 0.45), a tendency towards a decrease in General Stress (F(1,∞) = 2.76, p = 0.1, RTE 0.54) and no significant changes in the Success subscale (F(1,∞) = 0.01, p = 0.92, RTE 0.5).

The 1-week intervention induced no significant changes in the Short form-12 questionnaire (Physical Subscale: F(1,∞) = 0.31, p = 0.58, RTE 0.52; Mental Subscale: F(1,∞) = 1.88, p = 0.17, RTE 0.43). Also, the Mood Scale scores showed no changes over time (F(1,∞) = 0, p = 1, RTE 0.5).

The Felt Arousal Scale and the Feeling Scale did not show any significant changes over time, i.e., before versus after the ski tours. In addition, these values were correlated with physiological parameters, but again, there was no particular pattern (data not shown). All descriptive data of mental health are shown in Table A3.

## 4. Discussion

The aim of this feasibility study was to examine whether ski mountaineering following a TKA leads to muscle fatigue per se, or to the accumulation of even more fatigue during 1 week of recreational ski mountaineering. Additionally, assessing the feasibility of ski mountaineering as a leisure winter sports activity for patients with TKA was a target of this investigation. We found no significant decrease in muscle strength or functional abilities, such as gait and stair climbing, but a decrease in balance ability.

### 4.1. Consequences for Strength, Functional Abilities and Mood

Despite the reported decrease in quadriceps and hamstrings muscle strength after recreational ski mountaineering in healthy participants [16], we found no indication of muscle fatigue, expressed by strength loss in the operated leg. Neither a single day of ski mountaineering nor a complete week of repeated ski tours led to a decrease in muscle strength. In contrast to a prior study on ski mountaineering with healthy participants, we focused on a moderate individual exercise load based on the participant’s baseline cycle ergometry. All volunteers explicitly maintained their predefined heart rate limit during the study week and, therefore, overstrain was avoided. This accords with the results for the Borg scale, which indicated a moderate subjective exercise load. The results of cycle ergometry demonstrated an above-average fitness level based on the age-predicted exercise capacity [37]. Consequently, preventing overstrain and maintenance of a good fitness level might be indicators of an absence of muscle fatigue. In addition, the inclusion criteria required experience in ski mountaineering prior to and after the TKA implantation, to prevent additional exhaustion due to a lack of technical acumen in ski mountaineering. Moreover, the mountain guides chose routes with fewer kick turns in steep terrain, which might have been less demanding and, hence, less fatiguing. Eventually, albeit exclusively based on the observations of an accompanying member of the study team, the elderly study population seemed to appreciate the ski tours, engaging in small talk and enjoying a comfortable week outdoors in nature. This observation is lent support by the evidence that mental health and well-being increased during the week. The data revealed a tendency towards lower stress, as well as a significant increase in general well-being. In the context of physical and mental well-being, the natural environment is known to have a direct positive influence on physiological [38] and psychological health [39,40], due to stimulation of the visual, auditory and olfactory senses during exercise in nature [41,42]; however, these studies mainly related to nature in the summer season. The extent to which “white exercise”, which means exercise in a winter landscape, affects mental and physiological parameters is currently not known. However, our study indicates a positive influence here as well. Furthermore, according to Rogerson et al., the social interaction time during outdoors exercise is significantly greater than during indoor sports [43]. Moreover, Gladwell et al. provide evidence for an increased participation in physical activities in natural environments, through enhanced enjoyment, a raised frequency and more social interaction [44].

A significant decrease in stability over time was seen, as measured by the S3-Check MFTstability index, which is calculated based on the results of symmetry and sensorimotor performance. An explanation for this may be a deleterious effect of fatigue on sensorimotor function [45]. However, this result was only reflected in the two-legged stand (dynamic ground); the one-legged stand on the TKA leg (static ground) showed no statistical differences during the week. Regarding static ground, the sensorimotor system appeared to be able to compensate for tiredness, although the supportive area of the stand was smaller. However, dynamic balance control is especially important for ski mountaineering, because—particularly downhill—the ground and the position of the person change rapidly and continuously, where exteroceptive senses, such as the visual, auditive or vestibular systems, may be affected by ski wear, the surroundings or weather. Therefore, dynamic stability and good sensorimotor control are required. It would be of interest to investigate whether previous balance training relieves this fatigue effect, so that safety during ski mountaineering for people with TKA can be further increased.

Regarding the gait and Stair climb tests, no significant differences were found over the study week, nor between the affected and unaffected leg. An explanation for this could be that, for these two tests, interplay among strength, coordination and endurance occurs so that possible fatigue could be better compensated for.

### 4.2. Feasibility of Recreational Ski Mountaineering after TKA

It is well-known that both the number of ski mountaineers and the number of patients undergoing TKA is increasing [4,15]. Hence, exploring the feasibility of ski mountaineering after TKA is definitely justified. Several recommendations concerning different sports activities, such as hiking and downhill skiing, exist, but data on ski mountaineering are rare. Downhill skiing and hiking are categorised as “authorised sports activities with experience” after TKA [8]. Both activities are recommended for patients after TKA, while alpine skiing is also mentioned as a way to reduce strength deficits [6,13]. Reflecting the movement patterns required in these sports activities, it might be assumed that, in combination, they are similar to ski mountaineering However, the technical and physical demands of ski mountaineering are higher than those of alpine skiing and hiking, as it takes place under varying conditions and involves terrain handling, kick turns, and snowy conditions. Therefore, good technical skills and above average endurance are required. In the current study, we included only participants who met these criteria. In this context, we focused on the recommendations concerning a return to sports after TKA per se, and especially in terms of alpine skiing [8,14]. Considering this, and based on the study findings, we found no reason to recommend against ski mountaineering after TKA in comparably experienced patients. No significant decrease in muscle strength was detected, which might have indicated significant muscle fatigue during the study week, and there were no negative effects on functional abilities except for the aforementioned decline in stability. Hence, as muscle fatigue is known to increase injury risk [10], there might be no additional high risk for injuries for TKA patients with pre-existing skiing experience. This lends support to a prior study on the clinical and radiographic outcome of alpine skiers after 80 days of skiing within three years, where the authors did not observe any significant injury [46]. Furthermore, the Knee Society Score, measured before and 14 days after the investigation, remained stable. This indicates that there was no negative effect of recreational ski mountaineering on the prostheses of the study participants. Last but not least, we noted a positive effect on well-being and no complaints regarding exercise load, route selection, or the test battery. Future studies with larger samples are needed to confirm these results so that general recommendations can be made.

The study protocol used herein was carried out as planned; none of the patients reported adverse effects during measurements or ski trips. The participants showed high compliance and the ski mountaineering tours were well-tolerated. Nevertheless, a fall on descent (over a freshly snow-covered fence post) caused one dropout. This case was examined by experts and was seen as a ski accident that could have happened even without a prosthesis, reflecting the known risk of musculoskeletal injury associated with this sport [47]. The selected sequence of examinations (questionnaires–balance–gait–Stair climb test–strength) proved to be appropriate in practice.

The ski tours differed in terms of natural and planning-related conditions, reflecting a typical ski vacation week in the Alps. Relevant to our study was that the individual ski tours were standardized for each participant with respect to cardiovascular stress and duration, in order to be able to ensure an appropriate comparison of the participants.

### 4.3. Limitations

As this was a feasibility study that examined the practicability of recreational ski mountaineering for people with TKA for the first time, we included a relatively small number of participants. This investigation focused only on patients following a TKA with average skiing proficiency and experience in ski mountaineering in an open terrain before and after surgery. We selected this specific group of volunteers since we expected no ski-mountaineering-specific problems that we could not have excluded for less or unexperienced TKA patients. In addition, there are recommendations from alpine skiing, which state that it is allowed with experience [8,14], which was a further strong argument for our strict selection process. Another limitation might be the fact that we did not collect sufficient data on descents. This should be considered in a follow-up study to gain information on a possible exhaustion level of downhill skiing in open terrain. Furthermore, we did not consider the unaffected leg. This might be interesting in further investigations due to a possible compensation mechanism concerning strength of the TKA leg.

Based on our findings, a power calculation for the main parameter of strength, with five measurement time points, a power of β = 0.80, a significance level of 0.05, and a small expected effect size (f = 0.15), showed that 55 participants would be required in future studies. More reliable and generalizable recommendations could be made on similar issues, including recommendations regarding this sport for this population, with larger samples. Blinding is not possible with this study design, and another limitation was the gender ratio, such that a possible gender effect should be considered [1].

## 5. Conclusions

With regard to the study design, in compliance with the current recommendations concerning return to sports after TKA, this study provides an initial indication that recreational ski mountaineering for patients following TKA might be feasible, at least in persons with experience in skiing before surgery. Therefore, ski mountaineering as a leisure sports activity for this patient group should not be ruled out entirely. Actually, considering the low sample size and the selection of ski-experienced patients, making detailed recommendations is premature. A further investigation with a larger sample size, but employing the same design, might reveal findings allowing for solid recommendations for physicians to issue to their TKA patients. However, if patients tend to exercise on ski mountaineering on their own initiative and in spite of lacking recommendations, it is crucial to be well-prepared physically, to have sufficient experience in alpine skiing and above all to have clearance from the treating physician with respect to the condition of their knee replacement. Furthermore, in order to avoid overstrain, we recommend individually tailored endurance and strength training prior to the ski-mountaineering season.

## Figures and Tables

**Table 1:** Descriptive route information, heart rate and Borg Scale scores of recreational ski mountaineers.

	Ski Tour 1		Ski Tour 2		Ski Tour 3		Ski Tour 4		Ski Tour 5	
	t1		t2		t3		t5		t6	
	median	IQR	median	IQR	median	IQR	median	IQR	median	IQR
HR (bpm)	123.00	13.00	121.00	18.00	128.50	18.00	119.00	26.00	114.50	12.00
Time (min)	129.00	30.00	102.50	15.00	160.00	11.00	201.00	31.00	189.00	0.00
Ascent (m)	780.00 ^#^	-	715.00^#^	-	953.00	200.00	1037.00	35.00	998.00	0.00
Distance (m)	4400.00	200.00	3600.00^#^	-	4513.00	573.00	5380.00	373.00	5430.00	0.00
Borg Scale score	11.00	3.00	10.50	2.00	12.5	3.00	12.00	4.00	11.00	1.00

HR = average heart rate during ascent, Ascent = altitude difference during ascent, Distance = distance of ascent, ^#^ = constant values for all participants (no median/interquartile range (IQR) available).

**Table 2:** One-factor longitudinal analysis of Mobility LabGait parameters.

Mobility LabGait Parameters	F1-LD-F1			Relative Treatment Effects (RTEs)					
		F	*p*		UL	TKA		UL	TKA
Cadence	leg	0.03_(1,∞)_	0.87	leg × t1	0.41	0.44	leg × t4	0.52	0.41
	time	1.53_(2.34,∞)_	0.21	leg × t2	0.58	0.6	leg × t5	0.56	0.52
	leg × time	0.34_(2.34,∞)_	0.75	Leg × t3	0.49	0.45	leg × t6	0.5	0.51
Stride length	leg	0.02_(1,∞)_	0.88	leg × t1	0.5	0.54	leg × t4	0.41	0.45
	time	1.56_(2.39,∞)_	0.21	leg × t2	0.57	0.61	leg × t5	0.49	0.45
	leg × time	0.21_(2.39,∞)_	0.85	leg × t3	0.47	0.54	leg × t6	0.48	0.47
Double support time	leg	0.02_(1,∞)_	0.88	leg × t1	0.39	0.39	leg × t4	0.5	0.5
	time	0.9_(2.78,∞)_	0.44	leg × t2	0.51	0.5	leg × t5	0.53	0.5
	leg × time	0.01_(2.78,∞)_	1	leg × t3	0.58	0.56	leg × t6	0.53	0.5
Single limb support time	leg	1.12_(1,∞)_	0.29	leg × t1	0.64	0.59	leg × t4	0.47	0.51
	time	0.89_(2.58,∞)_	0.43	leg × t2	0.54	0.41	leg × t5	0.6	0.4
	leg × time	0.68_(2.58,∞)_	0.54	leg × t3	0.57	0.35	leg × t6	0.48	0.44
Stance	leg	0.58_(1,∞)_	0.44	leg × t1	0.46	0.28	leg × t4	0.45	0.63
	time	1.57_(2.56,∞)_	0.20	leg × t2	0.56	0.46	leg × t5	0.63	0.42
	leg × time	1.58_(2.56,∞)_	0.20	leg × t3	0.54	0.44	leg × t6	0.59	0.52
Swing	leg	0.32_(1,∞)_	0.57	leg × t1	0.56	0.73	leg × t4	0.56	0.38
	time	2.14_(2.69,∞)_	0.1	leg × t2	0.45	0.56	leg × t5	0.37	0.45
	leg × time	1.27_(2.69,∞)_	0.28	leg × t3	0.47	0.57	leg × t6	0.42	0.49
Elevation at midswing	leg	0.32_(1,∞)_	1	leg × t1	0.43	0.41	leg × t4	0.53	0.47
	time	1.6_(3.93,∞)_	0.17	leg × t2	0.56	0.48	leg × t5	0.54	0.44
	leg × time	0.1_(3.93,∞)_	0.98	leg × t3	0.66	0.57	leg × t6	0.48	0.44
Lateral step variability	leg	1.66_(1,∞)_	0.20	leg × t1	0.7	0.55	leg × t4	0.58	0.39
	time	1.64_(3.57,∞)_	0.17	leg × t2	0.38	0.55	leg × t5	0.59	0.54
	leg × time	1.41_(3.57,∞)_	0.23	leg × t3	0.49	0.38	leg × t6	0.56	0.28
Circumduction	leg	0_(1,∞)_	0.95	leg × t1	0.46	0.54	leg × t4	0.49	0.45
	time	1.31_(2.84,∞)_	0.27	leg × t2	0.59	0.56	leg × t5	0.5	0.42
	leg × time	0.53_(2.84,∞)_	0.65	leg × t3	0.51	0.51	leg × t6	0.48	0.5
Gait speed	leg	0.01_(1,∞)_	0.92	leg × t1	0.43	0.41	leg × t4	0.48	0.56
	time	2.01_(2.12,∞)_	0.13	leg × t2	0.66	0.66	leg × t5	0.5	0.42
	leg × time	0.39_(2.12,∞)_	0.69	leg × t3	0.45	0.57	Leg × t6	0.46	0.41

F1-LD-F1 = one-factor longitudinal analysis, UL = unaffected leg, TKA = leg with total knee arthroplasty.

## Abbreviations

Bf-SR	Mood Scale; German Befindlichkeits-Skala Revidierte Fassung
EBF 24 B/3	Shortened German Version of the Recovery-Stress Questionnaire for athletes
FAS	Felt Arousal Scale
FS	Feeling Scale
MFT	Multifunktionale Trainingsgeräte (Multifunctional Training Equipment)
OA	Osteoarthritis
RESTQ-Sport	Recovery-Stress Questionnaire for athletes
RTE	Relative treatment effect
SF12/SF36	Short form 12/36 Health Survey
TKA	Total knee arthroplasty

## Appendix A

**Table A1:** Descriptive Data of Strength and Balance.

	Arrival	Ski Tour 1	Ski Tour 2	Ski Tour 3	Ski Tour 4	Ski Tour 5
	Day 1	Day 2	Day 3	Day 4	Day 6	Day 7
Strength						
Concentric maximal voluntary contraction						
Concentric_Extensor_max (N·m)	116.00 ± 45.50	120.00 ± 57.50	117.00 ± 44.50	117.50 ± 49.50	107.00 ± 38.75	119.50 ± 46.75
Concentric_Flexor_max (N·m)	83.50 ± 43.00	81.00 ± 46.00	92.50 ± 43.50	82.00 ± 41.00	78.50 ± 45.75	87.50 ± 26.50
Concentric_Extensor_angle (degree)	58.50 ± 5.00	55.50 ± 4.00	57.50 ± 11.00	59.00 ± 12.00	58.00 ± 15.50	57.50 ± 4.75
Concentric_Flexor_angle (degree)	34.50 ± 12.50	33.50 ± 5.75	34.50 ± 8.75	36.00 ± 8.50	35.00 ± 7.50	35.50 ± 8.00
Isometric maximal voluntary contraction						
Isometric_Extensor_max (N·m)	134.50 ± 76.00	128.50 ± 87.00				132.00 ± 81.00
Isometric_Flexor_max (N·m)	70.50 ± 32.75	67.50 ± 27.75				70.00 ± 29.25
Strength endurance (SE)						
SE_Extensor_work (N·m)	790.00 ± 571.50	666.00 ± 580.50				805.50 ± 435.00
SE_Flexor_work (N·m)	724.00 ± 356.50	638.50 ± 393.25				733.50 ± 335.50
SE_Extensor_max (N·m)	81.50 ± 51.00	82.00 ± 54.50				85.00 ± 44.00
SE_Flexor_max (N·m)	68.50 ± 35.50	71.50 ± 36.50				67.00 ± 34.00
Balance (one and two legged stance)						
S3-Check MFT						
Stability Index (score)	4.85 ± 1.75	4.80 ± 1.05	5.25 ± 2.20	4.75 ± 1.15	5.40 ± 1.58	5.50 ± 1.75
Stability in relation to standard data (percent)	93.50 ± 38.25	95.50 ± 22.50	85.50 ± 49.00	95.50 ± 26.00	81.50 ± 32.50	79.00 ± 37.00
Sensorimotor Index (score)	4.40 ± 1.50	4.00 ± 0.95	4.00 ± 1.83	4.20 ± 1.50	3.60 ± 1.55	4.50 ± 1.43
Sensorimotor function in relation to standard data (percent)	102.00 ± 30.75	111.00 ± 20.50	112.00 ± 42.75	107.50 ± 29.75	120.00 ± 34.50	101.00 ± 32.25
Symmetry (deviation from horizontal plate position)	52.00 ± 8.50	50.00 ± 9.50	47.50 ± 23.00	47.00 ± 13.25	47.00 ± 22.25	51.50 ± 17.75
Deviation of 50	5.00 ± 3.75	4.00 ± 3.50	9.50 ± 14.25	5.50 ± 9.50	11.00 ± 12.75	8.00 ± 8.00
Mobility Lab balance parameter						
95% Ellipse Sway Area (m^2^/s^4^)	9.06 ± 6.07	6.23 ± 12.66	11.56 ± 12.68	10.64 ± 8.45	10.49 ± 12.68	7.89 ± 10.85

Data are represented as median ± IQR; SE = Strength endurance.

**Table A2:** Descriptive data of functional abilities (Gait parameters and Stair climb test).

	Arrival	Ski Tour 1	Ski Tour 2	Ski Tour 3	Ski Tour 4	Ski Tour 5
	Day 1	Day 2	Day 3	Day 4	Day 6	Day 7
Mobility Lab gait parameters						
CadenceTKA (steps per minute)	116.06 ± 9.44	120.70 ± 13.52	116.44 ± 14.29	113.59 ± 9.08	118.49 ± 10.20	116.82 ± 10.56
Cadence UL (steps per minute)	114.92 ± 10.06	119.36 ± 12.05	116.17 ± 16.85	117.87 ± 7.98	118.14 ± 11.03	117.93 ± 11.81
Stride length TKA (meter)	1.29 ± 0.10	1.32 ± 0.15	1.31 ± 0.15	1.27 ± 0.10	1.26 ± 0.09	1.26 ± 0.15
Stride length UL (meter)	1.27 ± 0.09	1.32 ± 0.19	1.28 ± 0.15	1.23 ± 0.12	1.27 ± 0.13	1.28 ± 0.17
Double support TKA (% of total gait cycle)	18.59 ± 5.04	21.13 ± 14.10	24.16 ± 13.16	18.89 ± 19.65	22.49 ± 11.67	20.13 ± 15.49
Double support UL (% of total gait cycle)	18.32 ± 7.06	22.02 ± 15.55	25.15 ± 15.12	18.67 ± 19.48	20.61 ± 15.75	20.90 ± 16.40
Single limb support TKA (% of total gait cycle)	41.50 ± 3.23	39.81 ± 3.24	39.28 ± 4.13	41.11 ± 6.69	39.82 ± 5.33	40.88 ± 4.36
Single limb support UL (% of total gait cycle)	41.72 ± 1.86	41.22 ± 2.11	42.21 ± 4.33	40.60 ± 5.63	41.50 ± 2.60	41.21 ± 3.89
Stance TKA (% of total gait cycle)	57.74 ± 2.13	58.49 ± 2.45	57.95 ± 5.52	60.89 ± 5.72	58.35 ± 3.66	58.68 ± 4.73
Stance UL (% of total gait cycle)	59.38 ± 2.64	60.18 ± 2.96	60.09 ± 3.51	58.74 ± 2.82	60.54 ± 4.96	58.99 ± 5.37
Swing TKA (% of total gait cycle)	42.26 ± 2.13	41.51 ± 2.45	42.05 ± 5.52	39.12 ± 5.72	40.83 ± 2.94	41.32 ± 4.73
Swing UL (% of total gait cycle)	40.63 ± 2.64	39.83 ± 2.96	39.91 ± 3.51	41.26 ± 2.82	39.46 ± 4.96	41.02 ± 5.37
Elevation at midswing TKA (centimeter)	1.72 ± 1.48	2.04 ± 2.31	2.66 ± 2.82	1.95 ± 1.80	1.77 ± 0.91	1.84 ± 1.59
Elevation at midswing UL (centimeter)	1.86 ± 1.47	2.41 ± 0.94	2.48 ± 1.02	2.22 ± 1.86	1.95 ± 1.28	2.06 ± 1.53
Lateral step variability TKA (centimeter)	3.61 ± 0.79	0.15 ± 1.29	3.29 ± 0.79	3.35 ± 1.13	3.47 ± 1.48	2.87 ± 0.90
Lateral step variability UL (centimeter)	4.03 ± 1.44	3.06 ± 1.14	3.35 ± 1.68	3.83 ± 0.85	3.86 ± 1.14	3.53 ± 1.85
Circumduction TKA (centimeter)	3.49 ± 1.41	3.62 ± 2.01	3.49 ± 1.88	3.20 ± 1.13	2.93 ± 2.15	3.17 ± 2.14
Circumduction UL (centimeter)	3.68 ± 1.59	3.37 ± 1.59	3.27 ± 1.16	3.39 ± 2.23	3.24 ± 1.59	3.27 ± 1.91
Gait Speed TKA (meter/second)	1.23 ± 0.10	1.30 ± 0.15	1.27 ± 0.17	1.28 ± 0.11	1.19 ± 0.14	1.18 ± 0.23
Gait Speed UL (meter/second)	1.23 ± 0.05	1.31 ± 0.18	1.21 ± 0.17	1.23 ± 0.16	1.23 ± 0.15	1.23 ± 0.23
Stair climb test						
Total time (seconds)	6.20 ± 1.22	6.04 ± 1.87	6.40 ± 2.35	5.99 ± 1.91	6.01 ± 1.23	6.08 ± 1.81

Data are shown as median ± IQR; UL = Unaffected leg, TKA = Leg with total knee arthroplasty

**Table A3:** Descriptive data of mental health.

	Arrival	Ski Tour 1	Ski Tour 2	Ski Tour 3	Rest Day	Ski Tour 4	Ski Tour 5	Departure
	Day 1	Day 2	Day 3	Day 4	Day 5	Day 6	Day 7	Day 8
VAS Well being		1.50 ± 3.00	3.00 ± 0.00	2.50 ± 3.00	3.00 ± 4.00	1.00 ± 3.00	2.00 ± 4.00	
Feeling Scale								
pre exercise		3.00 ± 1.00	3.00 ± 2.00	3.00 ± 2.00		2.00 ± 4.00	3.00 ± 3.00	
post exercise		4.00 ± 2.00	4.00 ± 2.00	4.00 ± 4.00		5.00 ± 2.00	4.00 ± 1.00	
Felt Arousal Scale								
pre exercise		3.00 ± 2.00	3.00 ± 1.00	3.00 ± 2.00		3.00 ± 1.00	3.00 ± 2.00	
post exercise		3.00 ± 2.00	3.00 ± 1.00	3.00 ± 1.00		3.00 ± 1.00	3.00 ± 2.00	
Short Form 12 Health Survey								
Physical Subscale	49.66 ± 7.99							49.63 ± 16.07
Mental Subscale	54.54 ± 8.98							58.46 ± 8.06
RESTQ-Sport								
Scale 1 General stress	0.00 ± 1.00							0.00 ± 0.50
Scale 2 Emotional stress	0.50 ± 1.00							0.50 ± 0.50
Scale 3 Social Stress	1.00 ± 0.50							0.50 ± 1.00
Scale 4 Conflicts/pressure	0.50 ± 1.00							1.00 ± 0.50
Scale 5 Fatigue	0.00 ± 1.00							0.00 ± 0.50
Scale 6 Lack of energy	1.00 ± 1.00							0.50 ± 0.50
Scale 7 Physical complaints	1.00 ± 1.00							1.50 ± 1.50
Scale 8 Success	3.00 ± 3.00							3.00 ± 4.00
Scale 9 Social recovery	3.50 ± 1.50							4.50 ± 3.00
Scale 10 Physical recovery	4.00 ± 1.00							4.50 ± 1.50
Scale 11 General well being	4.00 ± 1.00							5.00 ± 1.50
Scale 12 Sleep Quality	5.00 ± 2.50							5.00 ± 2.50
Mood Scale	6.50 ± 7.75							3.00 ± 11.00

Data are represented as median ± IQR; VAS = Visual Analogue Scale, RESTQ-Sport = Recovery-Stress Questionnaire for athletes.
